# Vaginal Microbiota Changes Caused by HPV Infection in Chinese Women

**DOI:** 10.3389/fcimb.2022.814668

**Published:** 2022-06-21

**Authors:** Yichan Zhang, Xiaolin Xu, Liqun Yu, Xingxian Shi, Min Min, Lijuan Xiong, Jia Pan, Yong Zhang, Peipei Liu, Guizhen Wu, Guolan Gao

**Affiliations:** ^1^ Savaid Medical School, University of Chinese Academy of Sciences, Beijing, China; ^2^ National Health Commission of the People’s Republic of China (NHC) Key Laboratory of Biosafety, National Institute for Viral Disease Control and Prevention, Chinese Center for Disease Control and Prevention, Beijing, China; ^3^ Department of Obstetrics and Gynecology, Aviation General Hospital of China Medical University, Beijing, China; ^4^ Department of Electrical and Computer Engineering, Johns Hopkins University, Baltimore, MD, United States; ^5^ Department of Obstetrics and Gynecology, Peking University International Hospital, The Eight Clinical Medical College, Beijing, China

**Keywords:** HPV infection, cervical intraepithelial neoplasia, vaginal microbiota, age, *L. gasseri–*dominated, community state types (CST)

## Abstract

Human papillomavirus (HPV) infection is one of the most common sexually transmitted diseases. After studying 602 unvaccinated Chinese women using 16S rRNA to detect cervical-vaginal microecology, we analyzed the relationship between HPV infection and vaginal microecology including 20 HPV types. In Chinese women, *L. gasseri–*dominated and *L. jensenii–*dominated clusters were significantly absence. Microbial alpha diversity was significantly higher in HPV-infected and cervical intraepithelial neoplasia (CIN)–diagnosed groups than in healthy control group. Certain bacteria were associated with HPV infection and CIN, including *Streptococcus*, *Prevotella*, *Chlamydia*, *Bifidobacterium*, *Ralstonia*, and *Aerococcus.* With the development of disease, the proportions of community state type III (CST-III) and CST-IV-B gradually increased, whereas the proportions of CST-I and CST-IV-A gradually decreased. In addition, age was an influential factor for HPV infection. With aging, the probability of HPV infection and the proportion of CST-IV-B increase. In conclusion, our study was a large cross-sectional study that evaluated the relationship between vaginal microbiota and HPV infection, and brought essential comparable data.

## Introduction

**Cervical cancer (CC)** is a malignant tumor originating in the cervix. **Cervical intraepithelial neoplasia (CIN)** is a precancerous lesion closely related to invasive CC that includes cervical atypical hyperplasia and carcinoma *in situ* (Avilés-Jiménez et al., 2017). On the basis of the extent of atypical proliferative cells in the cervical squamous epithelium, CIN can be classified into grades 1, 2, and 3 (Moodley et al., 2003). **Human papillomavirus (HPV)**, a major CC pathogenic factor, belongs to the family *Papillomaviridae* and the genus *Papillomavirus* (Piersma, 2011). HPVs involved in reproductive tract infections are divided into two types: high-risk type (HR-HPV) and low-risk type (LR-HPV). HR-HPV infection is an internationally recognized pathogenic factor for CC (Chen et al., 2019). LR-HPV is related to mild squamous epithelial lesions and genitourinary warts. HPV can be transmitted through sexual contact, non-sexual contact, and vaginal delivery (Di Paola et al., 2017).

**Vaginal microbiota (VMB)** are the vaginal microorganisms present in a woman of reproductive age. VMB are normally dominated by *Lactobacillus* spp. that breaks down glycogen to secret lactic acid, which makes the pH of the vaginal environment acidic (Borgogna et al., 2020). According to Gajer et al, the vaginal microbiome is classified into six community state types (CST) based on differences in species composition and their relative abundance (Gajer et al., 2012). CST-I, CST-II, and CST-III are dominated by *L. crispatus*, *L. gasseri*, and *L. iners*, respectively. The CST-IV-A is generally characterized by modest proportions of either *L. crispatus*, *L. iners*, or other *Lactobacillus* spp., along with low proportions of various species of strictly anaerobic bacteria. The CST-IV-B has higher proportions of *Prevotella*, *Parvimonas*, *Sneathia*, *Gardnerella*, *Mobiluncus*, and *Peptoniphilus* plus several other taxa (Gajer et al., 2012).

Previous studies have shown that the vaginal microbiome is an important part of the female vaginal microenvironment that maintains vaginal health and is the first line of defense against sexually transmitted infections (Mitra et al., 2016). In a healthy state, the vaginal microbiome forms a bacterial membrane on the surface of the cervical and vaginal mucosa and secretes lactic acid, bacteriocin, and a biological surfactant, which prevents adhesion and promote autophagy and clearance of pathogenic bacteria (Borgdorff et al., 2016; Amabebe and Anumba, 2018). Factors like HPV infection can lead to abnormal changes in the vaginal microenvironment and predominant non–*Lactobacillus* spp., which secrete short-chain fatty acids (SCFAs) that destroy the original protection mechanism of VMB (Delgado-Diaz et al., 2020; Li et al., 2020). HPV destroys the original balance of VMB by inhibiting the growth of *Lactobacillus* spp. and increasing the diversity of species especially strict anaerobic bacteria that produce enzymes and metabolites (Norenhag et al., 2020; Andrade Pessoa Morales et al., 2021). This destroys the cervical epithelial barrier and promotes HPV entry (Boskey et al., 2001). These bacteria also act on several cellular pathways that enable sustained HPV infection and subsequent disease development (Di Pietro et al., 2017; Murall et al., 2019).

Studies on the vaginal microbiology of Chinese women lag behind those of other countries and the current research is still in the observation stage (Chen et al., 2020; Mei et al., 2022). These studies have focused mainly on specific physiological states such as pregnancy, perimenopause, and those diagnosed with cervical cancer and therefore have small sample sizes (Chao et al., 2019; Wu et al., 2020; Zhai et al., 2020). Our study analyzed large-scale data of Chinese women of all ages ranging from those with HPV infection to those with cervical cancer. It paves the way for large follow-up studies.

## Methods

### Study Population

All study participants provided written informed consent according to good clinical practice guidelines. The study was approved by the Aviation General Hospital Ethics Committee, and the certificate number was 2021-KY-01-02. The inclusion criteria were as follows: a) age range from 20 to 70 years; b) willing to cooperate during the collection of cervical and vaginal samples; c) voluntarily participate and sign written informed consent; and d) no plan to move out of Beijing within 2 years and willing to cooperate with follow-up. The exclusion criteria were as follows: a) pregnancy; b) vaccinated against HPV; c) sexual activity, vaginal irrigation, or drug application performed within 48 h before sampling; d) diagnosis with tuberculosis, hepatitis B virus (HBV), hepatitis C virus (HCV), sexually transmitted infections (HIV, *Treponema pallidum*, etc.), and other infectious diseases; e) diagnosed with bacterial vaginosis (BV), vaginal candidiasis, *Trichomonas vaginalis* (TV), *Neisseria gonorrhoeae*, *Chlamydia trachomatis*, etc.; and f) patients with immune system disorders or chronic diseases (diabetes mellitus, hypertension, and coronary heart disease). Participants were also asked and collected questions related to HPV infection, including number of pregnancy and gestation, number of recent sexual partners, whether they smoked, whether they used vaginal douche, whether they used contraception, and methods of contraception. Details could see **Supplementary Figure 6**.

### Sample Collection and Storage

After cervical and vaginal samples were collected, each cervical brush (Huaxia Ruitai Plastic Industry Company, Jiangsu, China) was rinsed in a vial of normal saline solution, placed in a temporary sample transport box (0°C), and transferred to a 4°C refrigerator as soon as possible. The samples could be stored at 4°C for a month (Borgdorff et al., 2016).

### DNA Extraction

Metagenomic DNA samples were extracted by using the Fast DNA SPIN extraction kits (QIAamp PowerFecal Pro DNA Kit, Qiagen, China) (Li et al., 2020). Bacterial DNA was extracted from the secretions by filtration column and rapid centrifugation. The interference of human tissue DNA was eliminated and 100 μl of DNA sample was obtained. It was possible to store the DNA samples at −80°C for a long time. The DNA samples were sent to Nuohe Zhiyuan Technology Company (Beijing, China) for sequencing analysis of 16S amplicon.

### 16S rRNA Gene Sequencing

After genomic DNA extraction, sterile water was used to dilute the sample to 1 ng/μl, using specific primers with barcode. The Phusion High-Fidelity PCR Master Mix with GC Buffer (New England Biolabs, France) was used for PCR. The TruSeq DNA PCR-free Sample Preparation Kit (Illumina Inc., San Diego, USA) was used for library construction. The constructed library was quantified by Qubit and Q-PCR and then sequenced using the NovaSeq high-throughput sequencing platform (PE250) (Illumina Inc., San Diego, USA) (Chen et al., 2020; Mei et al., 2022).

### Sequencing Data Analysis

After amputation of the barcode and primer sequences, reads of each sample were joined using FLASH (V1.2.7, http://ccb.jhu.edu/software/FLASH/), and the Raw Tags were obtained. The Raw Tags need to go through strict filtering to get high-quality Clean Tags data. The Reference Qiime (V1.9.1, http://qiime.org/scripts/split_libraries_fastq.html) Tags quality control process was used to get the final Effective Tags using the Uparse algorithm (Uparse v7.0.1001, http://www.drive5.com/uparse/) for all samples (http://drive5.com/uparse/). By default, sequences are clustered into operational taxonomic units (OTUs) with 97% identity. The OTU sequences were annotated by species using the Mothur method and SSUrRNA database of SILVA138 (http://www.arb-silva.de/). We used the MUSCLE software (version 3.8.31, http://www.drive5.com/muscle/) for rapid multiple sequence alignment (Chen et al., 2020; Mei et al., 2022).

The Qiime software (Version 1.9.1) was used to calculate OTUs, Chao1, Shannon, Simpson, abundance-based coverage estimator (ACE), Good’s coverage, and PD_whole_tree indices, and R software (version 2.15.3) was used to map dilution curves, rank abundance curves, species accumulation curves, and principal coordinates analysis (PCoA) plots. The LEfSe analysis was performed using LEfSe software, and the default value of the linear discriminant analysis (LDA) score was 4.

### HPV Genotyping and Pathology Results

Women with cervical lesions detected by colposcopy were biopsied for pathology. The pathology results and CIN diagnosis were obtained from the Aviation General Hospital of China Medical University. The Hybrid Capture 2 assay (HC2) was used to detect HR-HPV types and low-risk types. Women who were positive for HR-HPV also accepted TCT (Thinprep Cytologic Test) to define the cytological lesion. The clinician will examine the cervix under a colposcope and will perform cervical biopsy for suspected lesions. Immunohistochemical methods were used to classify cervical tissues.

### Statistical Analysis

The Wilcoxon rank sum test was employed when comparing microbial alpha diversities between two groups and more than two groups. The Benjamini–Hochberg correction was conducted to decrease the false discovery rate for multiple tests (Mitra et al., 2020). The association between microbiota community structure and HPV infection was analyzed by multivariable logistic regression and adjusted for age and presented as odds ratio and 95% confidence interval (CI), with a 95% CI not including an OR of 1 indicating statistically significant differences. The relationship between HPV infection, vaginal microbiota, and age was compared by χ^2^ test (Agostinis et al., 2019). All statistical analyses were calculated using SPSS version 25 (IBM, New York, NY) and R software (V3.6.3), and a *P*-value less than 0.05 was considered statistically significant.

## Results

### Participant Characteristics in the Study Cohort

The participants of this study were Chinese women who visited the Aviation General Hospital of China Medical University from November 2020 to May 2021. We collected 602 samples including 458 samples from the gynecological clinic and 144 samples from the medical center. After ruling out unqualified samples, 356 effective samples were used in the study. These subjects were divided into three groups that included a healthy control (HC) group (n = 113), an HPV-infected group (n = 159), and a CIN-diagnosed group (n = 84). In the HPV group, participants were also grouped into those with a simple HPV (HPV-S) infection (n = 107) and multiple HPV (HPV-M) infections (n = 52). The CIN-diagnosed participants were grouped into CIN1 group (n = 47), CIN2 group (n = 24), and CIN3 group (n = 13) (**Table 1**).

**Table 1:** The characteristics of all included women.

	CST-I (N = 102)	CST-II (N = 2)	CST-III (N = 111)	CST-IV-A (N = 40)	CST-IV-B (N = 99)	CST-V (N = 2)	Total (N = 356)	P-value (χ2 test)
**HC**	34 (30.09%)	2 (1.77%)	28 (24.78%)	20 (17.70%)	28 (24.78%)	1 (0.88%)	113	0.137
**HPV**	46 (28.93%)	0 (0.00%)	54 (33.96%)	11 (6.92%)	47 (29.56%)	1 (0.63%)	159	
**CIN**	22 (26.19%)	0 (0.00%)	29 (34.52%)	9 (10.71%)	24 (28.57%)	0 (0.00%)	84	
**CIN1**	13 (27.66%)	0 (0.00%)	17 (36.17%)	5 (10.64%)	12 (25.53%)	0 (0.00%)	47	0.988
**CIN2**	6 (25.00%)	0 (0.00%)	7 (29.17%)	3 (12.50%)	8 (33.33%)	0 (0.00%)	24	
**CIN3**	3 (23.08%)	0 (0.00%)	5 (38.46%)	1 (7.69%)	4 (30.77%)	0 (0.00%)	13	
**HPV-S**	29 (27.10%)	0 (0.00%)	37 (34.58%)	8 (7.48%)	32 (29.91%)	1 (0.93%)	107	0.9
**HPV-M**	17 (32.69%)	0 (0.00%)	17 (32.69%)	3 (5.77%)	15 (28.85%)	0 (0.00%)	52	

**Figure 1:**
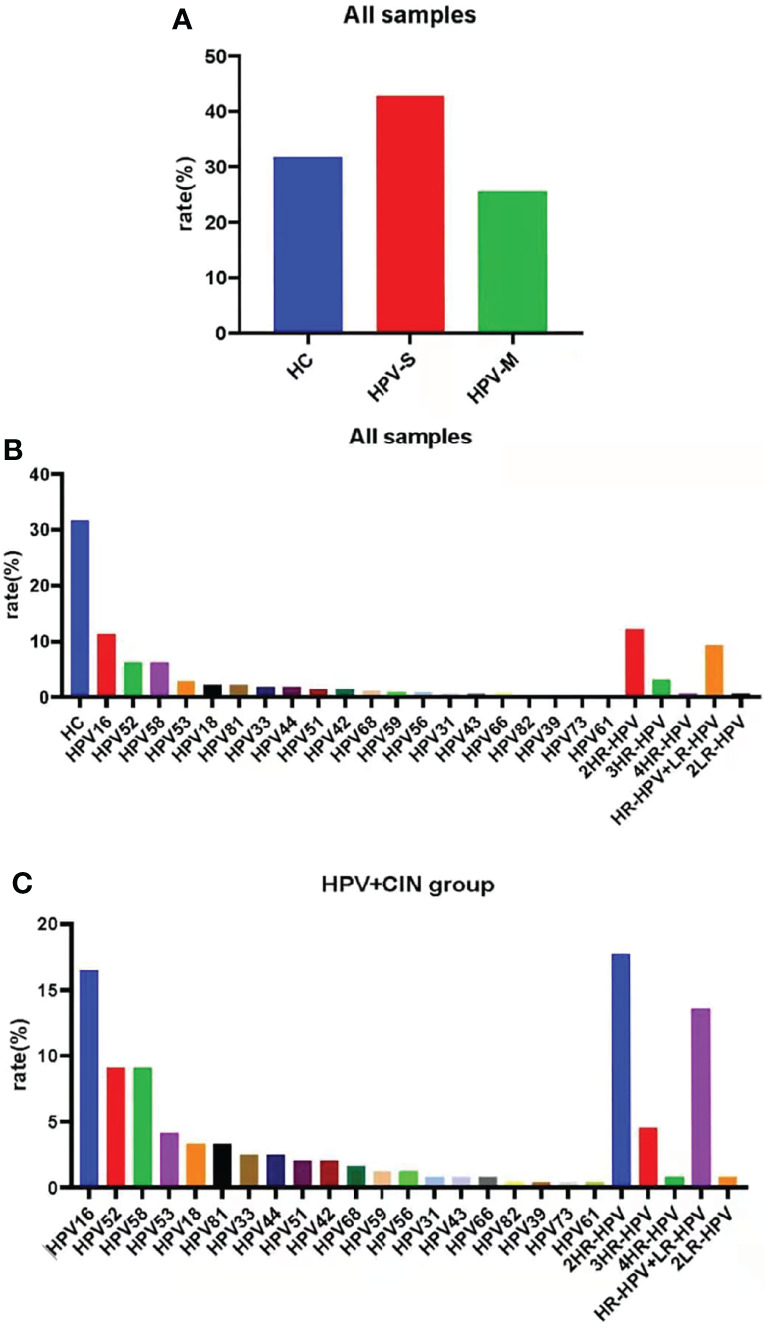
Samples were classified by HPV subtypes. **(A)** The percentage of HC group, simple-infection group, and multiple-infection group in all samples. **(B)** The percentage of special HPV types in all samples. **(C)** HPV prevalence in the HPV-infected and CIN-diagnosed groups. The top HPVs are HPV16, 52, 58; 2HR-HPV; and HR-HPV + LR-HPV.

In all samples, the percentage of HPV negative samples was 31.74% (n = 113), the percentage of HPV-S infections was 42.70% (n = 152), and the percentage of HPV-M infections was 25.56% (n = 91). The top three HPV types were 2HR-HPV infection (n = 43, 12.08%), HPV16 (n = 40, 11.24%), and HR-HPV plus LR-HPV infection (n = 33, 9.27%) (**Figures 1A, B**; **Supplementary Table 1**).

Except for HC group, samples in the HPV infection and CIN-diagnosed groups were also divided into those with HPV-S infection (n = 152, 62.55%) and HPV-M infection (n = 91, 37.45%). In the participants with HPV-S infection, the top three HPV types were HPV16 (n = 40, 16.46%), HPV52 (n = 22, 9.05%), and HPV58 (n = 22, 9.05%); whereas, in the participants with HPV-M infections, the top three HPV types were 2HR-HPV infection (n = 43, 17.70%), HR-HPV plus LR-HPV infection (n = 33, 13.58%), and 3HR-HPV infection (n = 11, 4.53%) (**Figure 1C**; **Supplementary Table 2**).

### Histogram of Relative Abundance of Species and Rank Abundance Curve

**Figure 2:**
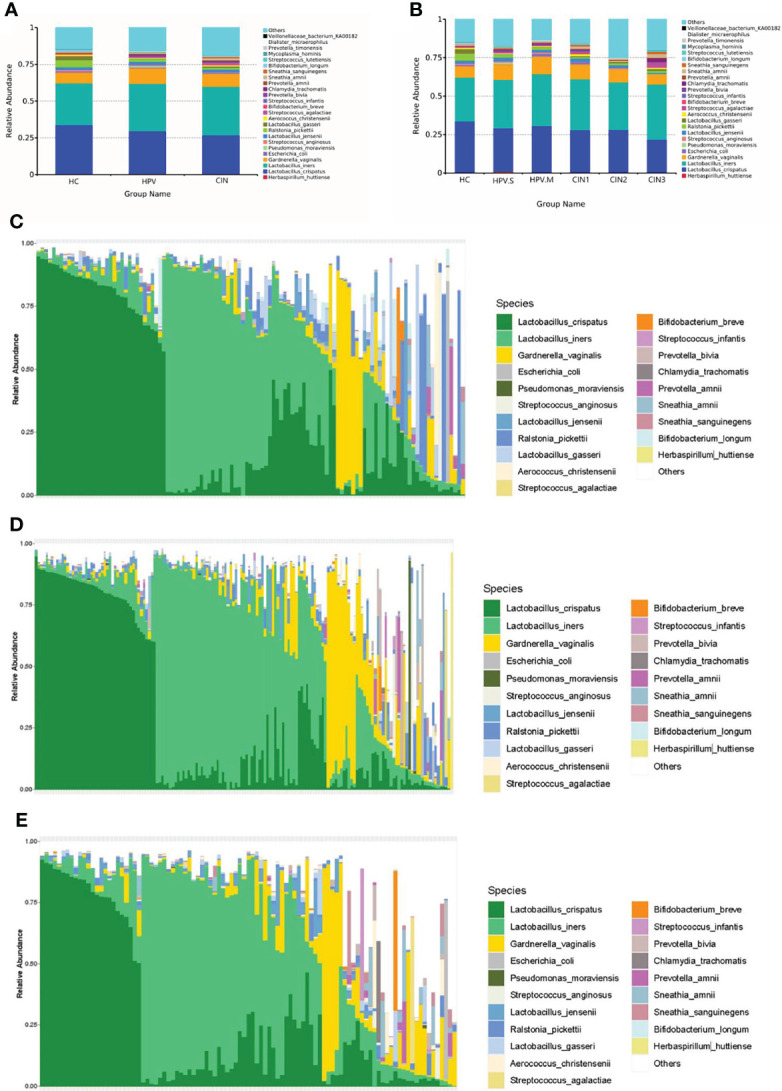
Histogram of relative abundance of species. **(A)** Vaginal microbiota at the species level in HC, HPV, and CIN groups. **(B)** Vaginal microbiota at the species level in HC, HPV-S, HPV-M, CIN1, CIN2, and CIN3 groups. **(C)** Vaginal microbiota at the species level from healthy control group women. **(D)** Vaginal microbiota at the species level from HPV-infected group women. **(E)** Vaginal microbiota at the species level from CIN group women.

There were changes of species in the HC, HPV, and CIN groups. The proportion of *L. crispatus* was decreased (HC: 33.61%; HPV: 28.96%; CIN: 26.77%), whereas that of *L. iners* was increased (HC: 28.65%; HPV: 32.36%; CIN: 33.15%). The proportion of *Lactobacillus*-dominated categories was decreased (HC: 67.13%; HPV: 64.08%; CIN: 62.8%) and that of non-*Lactobacillus–*dominated categories was increased (HC: 32.87%; HPV: 35.92%; CIN: 37.2%) (**Figures 2A–E**).

The greater the span of the curve along the horizontal axis, the higher the species richness was. The species richness in the HPV and CIN groups was higher than that in the HC group. In the vertical direction, the smoothness of the curve reflects the uniformity of species in the sample. There was no significant difference between the three groups, and the species distribution was even (**Supplementary Figure 1A**).

### Comparison of Samples Diversity

**Figure 3:**
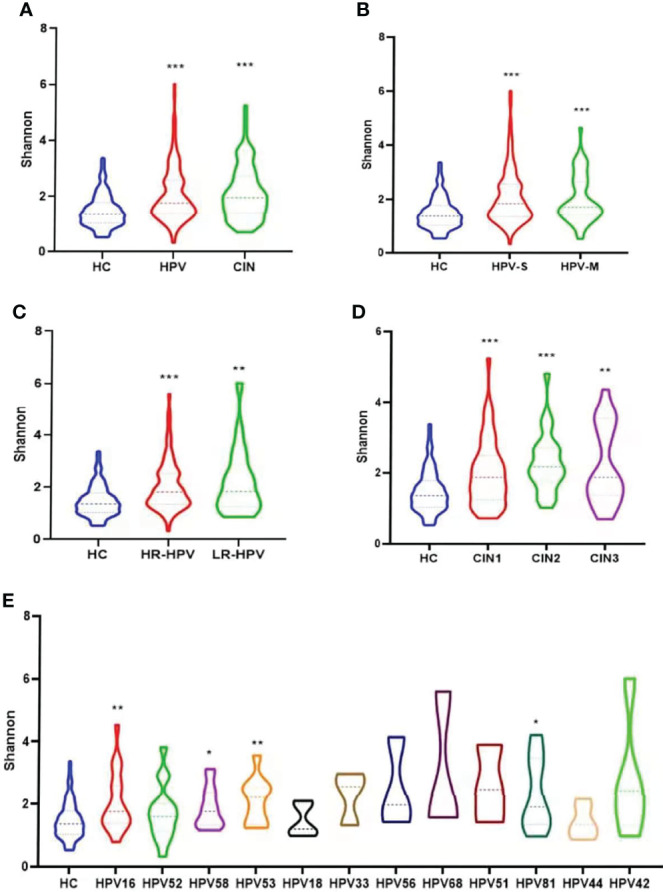
Microbial alpha diversity analysis based on Shannon index. **(A)** Microbiota diversity (Shannon) comparison among groups of healthy control women, HPV-infected women and CIN-diagnosed women (Wilcoxon one-side test; ***p < 0.0001). **(B)** Microbiota diversity comparison of healthy control group, and groups infected with single and multiple HPV types (Wilcoxon one-side test; ***p < 0.0001). **(C)** Microbiota diversity comparison of healthy control group, and groups infected with high-risk (HR) and low-risk (LR) HPV types (Wilcoxon one-side test; **p < 0.005 and ***p < 0.0001). **(D)** Microbiota diversity comparison of healthy control group, and groups diagnosed with CIN1, CIN2, and CIN3 (Wilcoxon one-side test; **p < 0.005 and ***p < 0.0001). **(E)** Microbiota diversity among participants infected with different HPV types in comparison with healthy women (Wilcoxon one-side test; *p < 0.05 and **p < 0.005).

The Shannon, Chao1, and Faith’s diversity analyses all revealed significantly higher microbial alpha diversity in the HPV and CIN groups than that in the HC group (Wilcoxon one-side test; HPV: *P* < 0.0001 for Shannon, *P* < 0.0001 for Chao1, and *P* < 0.0001 for Faith’s diversity; CIN: *P* < 0.0001 for Shannon, *P* < 0.0001 for Chao1, and *P* < 0.0001 for Faith’s diversity; **Figure 3A**; **Supplementary Figures 2A, 3A**).

Women with HPV-S infection, HPV-M infection (Wilcoxon one-side test; HPV-S: *P* < 0.0001 for Shannon, *P* < 0.0001 for Chao1, and *P* < 0.0001 for Faith’s diversity; HPV-M: *P* < 0.0001 for Shannon, *P* < 0.0001 for Chao1, and *P* < 0.0001 for Faith’s diversity; **Figure 3B**; **Supplementary Figures 2B, 3B**), HR-HPV, and LR-HPV had higher alpha diversity than women in the HC group (Wilcoxon one-side test; HR-HPV: *P* < 0.0001 for Shannon, *P* < 0.0001 for Chao1 and *P* < 0.0001 for Faith’s diversity; LR-HPV: *P* = 0.009 for Shannon, *P* = 0.003 for Chao1 and *P* = 0.005 for Faith’s diversity; **Figure 3C**; **Supplementary Figures 2C, 3C**). The alpha diversities of VMB in different CIN groups were significantly higher than that in the HC group (Wilcoxon one-side test; CIN1: *P* < 0.0001 for Shannon, *P* < 0.0001 for Chao1, and *P* = 0.006 for Faith’s diversity; CIN2: *P* < 0.0001 for Shannon, *P* = 0.001 for Chao1, and *P* = 0.002 for Faith’s diversity; CIN3: *P* = 0.003 for Shannon, *P* = 0.044 for Chao1, and *P* < 0.0001 for Faith’s diversity; **Figure 3D**; **Supplementary Figures 2D, 3D**).

As shown in **Figure 3E**, in the groups of HPV types with enough women in the group for analysis (n > 3), women infected with HPV16, 58, 53, and 81 had significantly higher diversity compared with the HC group in the Shannon analysis (Wilcoxon one-side test; HPV 16: *P* = 0.005; HPV58: *P* = 0.017; HPV 53: *P* = 0.006; HPV 81: *P* = 0.043; **Figure 3E**). The HPV16, 58, and 81 in the Chao1 analysis (Wilcoxon one-side test; HPV 16: *P* = 0.014; HPV58: *P* = 0.001; HPV 81: *P* = 0.011; **Supplementary Figure 2E**) and HPV16, 58, 53, 56, 68, and 81 in Faith’s diversity analysis were the HPV types showing significantly higher diversity compared with the HC group (Wilcoxon one-side test; HPV 16: *P* < 0.0001; HPV58: *P* = 0.001; HPV 53: *P* = 0.049; HPV 56: *P* = 0.022; HPV 68: *P* = 0.038; HPV 81: *P* = 0.007; **Supplementary Figure 3E**).

### Bacterial Taxa Analysis

**Figure 4:**
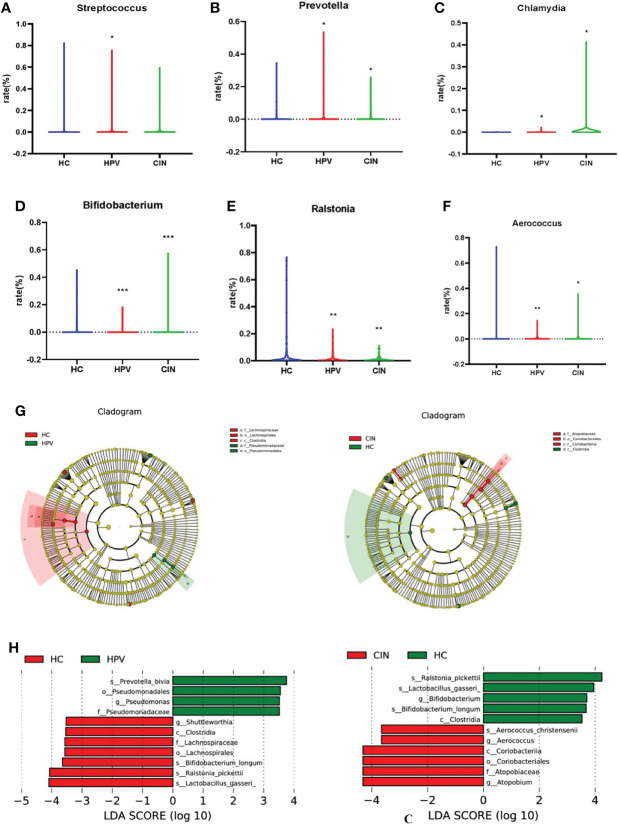
Bacterial Taxa Analysis. **(A)** *Streptococcus* was significantly higher in HPV-infected group than HC group. **(B, C)** *Prevotella* and *Chlamydia* were the bacterial species that significantly higher in HPV- infected group and CIN-diagnosed group than HC group. **(D, E)** *Bifidobacterium* and *Ralstonia* were significantly lower in HPV-infected group and CIN-diagnosed group than HC group. **(F)** *Aerococcus* was lower in HPV-infected women and higher in CIN-diagnosed women compared with HC women. **(G)** Cladogram of the LEfSe analysis of the vaginal microbiota in the three study groups. The microbial compositions were compared at different evolutionary levels. **(H)** LDA scores obtained from the LEfSe analysis of the vaginal microbiota in the different groups. An LDA effect size of >2 was used as a threshold for the LEfSe analysis. LDA, linear discriminant analysis; LEfSe, LDA effect size analysis. Wilcoxon one-side test; *P < 0.05 and **P < 0.005. HC, healthy control group; HPV, HPV-infected group; CIN, CIN-diagnosed group.

Certain bacterial species were related to HPV infection and CIN. We compared the relative abundance of all the bacteria from samples to identify potential bacterial biomarkers for HPV infection and CIN. From statistical analysis on microbiota taxonomy, we observed that *Streptococcus*, *Prevotella*, and *Chlamydia* were significantly more prevalent in HPV-infected women than in HC women (Wilcoxon one-side test; *Streptococcus*: *P* = 0.016; *Prevotella*: *P* = 0.013; and *Chlamydia*: *P* = 0.010; **Figures 4A–C**). *Prevotella* and *Chlamydia* were more prevalent in the women with CIN than the HC women (Wilcoxon one-side test; *Prevotella*: *P* = 0.031; *Chlamydia*: *P* = 0.038; **Figures 4B, C**). *Bifidobacterium* and *Ralstonia* were less prevalent in HPV-infected and CIN-diagnosed women than the HC women (Wilcoxon one-side test; *Bifidobacterium*: *P* < 0.0001; *Ralstonia*: *P* = 0.003; **Figures 4D, E**). *Aerococcus* was less prevalent in the HPV group than that in the HC group; whereas *Aerococcus* was more prevalent in the CIN group than that in the HC group (Wilcoxon one-side test; HPV group: *P* = 0.001; CIN group: *P* = 0.032; **Figure 4F**).

The LEfSe analysis revealed that compared with the HC group, the abundance of *Prevotella bivia* and *Pseudomonadales* was decreased whereas the abundance of *Shuttleworthia*, *Clostridia Lachnospiraceae*, *Bifidobacterium*, *Ralstonia pickettii*, and *Lactobacillus gasseri* was significantly increased in the HPV group (**Figure 4G**). The abundance of *Aerococcus*, *Coriobacteriia*, and *Atopobium* was significantly increased in the CIN group compared with the HC group, whereas the abundance of *Ralstonia*, *Bifidobacterium*, *L. gasseri*, and *Clostridia* was decreased (**Figure 4H**).

### Vaginal Microbiota in Chinese Women

**Figure 5:**
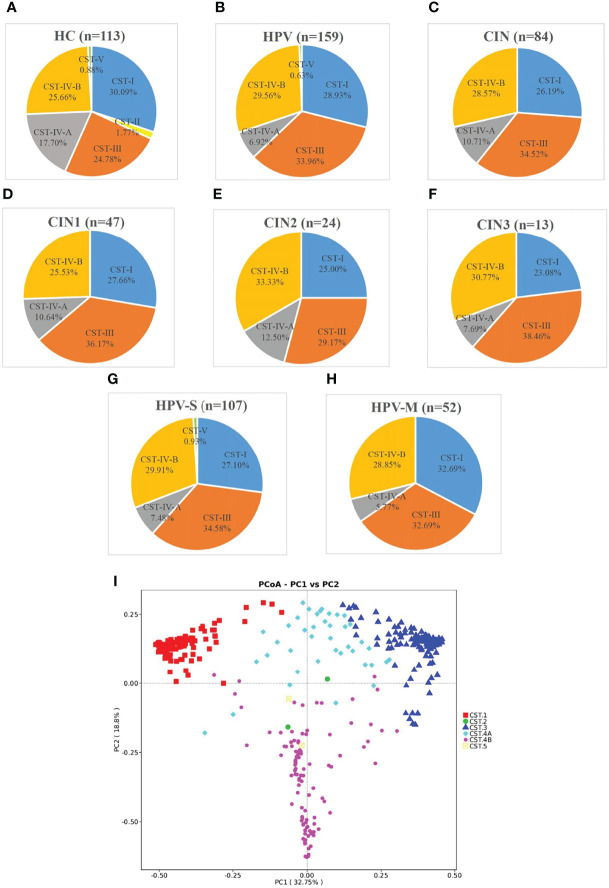
Samples were classified by CST and principal coordinates analysis (PCoA) of microbiota data based on Bray–Curtis distance of vaginal microbiota. **(A–H)** According to community state types (CSTs), the VMB of women was divided into I-V subtypes (n, the number of samples; HC, healthy control group; HPV, HPV-infected group; CIN, CIN-diagnosed group; HPV-S, HPV simple infection group; HPV-M, HPV multiple infection group; CIN1, CIN1 group; CIN2, CIN2 group; CIN3, CIN3 group). **(I)** Principal coordinates analysis (PCoA) of microbioal species data based on Bray–Curtis distance matric demonstrated six main vaginal microbiota clusters.

In all the samples, the participants expressing the CST-II types characterized by a dominance of *L. gasseri* were few. In the HC group, CST-II only has two participants (1.77%), whereas the HPV and CIN groups had no participants with CST-II (**Figure 5C**). The PCoA based on the Bray–Curtis index demonstrated that the samples were mainly separated into modest proportions of either *L. crispatus*, *L. iners*, or other *Lactobacillus* spp and non-*Lactobacillus*–dominated categories (**Figure 5I**).

With the progression of the disease, the distribution of CST changed significantly. The percentage of CST-I decreased and was 30.09% in the HC group, 28.93% in the HPV group, and 26.19% in the CIN group. The trend of CST-IV-A was also similar and with 17.70% in the HC group, 6.92% in the HPV group, and 10.71% in the CIN group (**Figures 5A–C**). On the contrary, the percentages of CST-III and CST-IV-B were increased. The percentage of CST-III was 24.78% in the HC group, 33.96% in the HPV group, and 34.52% in the CIN group. The percentage of CST-IV-B was 25.66% in the HC group, 29.56% in the HPV group, and 28.57% in the CIN group (**Figures 5A–C**).

We found that compared with the HC group, the trend of changes in the HPV and CIN groups was similar. The percentages of CST-I in the CIN1, CIN2, and CIN3 subgroups were 27.66%, 25.00%, and 23.08%, respectively. The percentages of CST-IV-A in the CIN1, CIN2, and CIN3 subgroups were 10.64%, 12.50%, and 7.69%, respectively, whereas those for the HPV-S and HPV-M subgroups were 7.48% and 5.77%, respectively. In contrast, the percentage of CST-III and CST-IV-B in the CIN1, CIN2, and CIN3, HPV-S, and HPV-M subgroups were increased compared with the HC group (CST-III: 36.17%, 29.17%, 38.46%, 34.58%, and 32.69%; CST-IV-B: 25.53%, 33.33%, 30.77%, 29.91%, and 28.85%) (**Figures 5D–H**). The HPV-M infection group showed 32.69% of CST-I, which was higher than that in the HC group (**Figures 5A, H**).

The cervical and vaginal microbiota were distributed into six clusters using heatmap analysis at the species level (**Supplementary Figure 4**). *L. crispatus* was predominant in cluster I, *L. gasseri* was predominant in cluster II, and *L. iners* was predominant in cluster III. Modest proportions of either *L. crispatus*, *L. iners*, or other *Lactobacillus* spp were dominant in cluster IV-A, and non-*Lactobacillus*–dominated categories such as *Gardnerella vaginalis*, *Prevotella amnii*, *Sneathia amnii*, *Sneathia sanguingens*, *Prevotella timonensis*, and *Ralstonia pickettii* were abundant in cluster IV-B (**Supplementary Figure 4**).

The six groups, which included HC, HPV-S, HPV-M, CIN1, CIN2, and CIN3, expressed the results of PCoA based on the Bray–Curtis index, and no clear separation was observed in the PCoA plot among each group, indicating no significant differences among these groups (**Supplementary Figure 5**).

### Age and Vaginal Microbiota Showed Correlation to HPV Infection and Cervical Lesions

We conducted logistic regression analysis on possible risk factors for HPV infection including age, CST types, number of gestation and pregnancies, contraception situation, IUD (Intrauterine Device) implant, number of sexual partners, smoking characteristics, and usage of vaginal douche, which were collected in the questionnaire. As shown in **Supplementary Figure 6**, we found that age (OR = 1.074; 95% CI = 1.027~1.122; *P* = 0.002) and contraception (OR = 3.796; 95% CI = 1.110~12.982; *P* = 0.034) were risk factors for HPV infection, whereas CST types (OR = 0.744; 95% CI = 0.592~0.933; *P* = 0.011), number of pregnancy (OR = 0.237; 95% CI = 0.098~0.577; *P* = 0.001), number of sexual partners (OR = 0.402; 95% CI = 0.246~0.655; *P* < 0.0001), and IUD implant (OR = 0.344; 95% CI = 0.172~0.687; *P* = 0.003) were protective factors for HPV infection. In addition, we also conducted logistic regression analysis on possible risk factors for CIN, which included HPV-S infection and HPV-M infection, and we just found that HPV infection was a risk factor for (OR= 4.137; 95% CI = 2.720~6.291; *P* < 0.0001) CIN. We chose HPV-S and HPV-M and special HPV types, with CIN 1/2/3, by using the χ^2^ test, and we did not find significantly change (**Supplementary Table 4**) .

**Figure 6:**
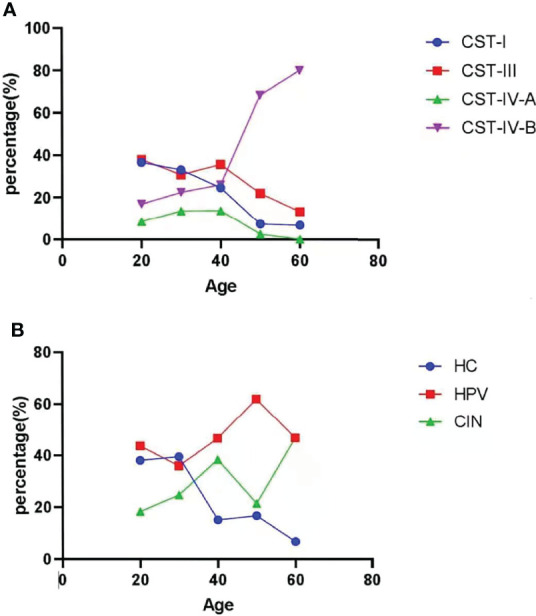
HPV infection and CST subtypes based on age. **(A)** Different CST subtypes in all samples according to age. **(B)** HPV prevalence and CIN-diagnosed in all samples according to age.

Logistic regression analysis showed that age was significantly associated with the risk of being HPV-infected (**Supplementary Figure 6**). Subjects were divided into five groups by age group of 10 years, covering women from 20 to 69 years of age. About HPV infection and cervical lesions, with age, the percentage of healthy subjects decreased, whereas the percentage of HPV-infected subjects and cervical lesion subjects obviously increased (**Figure 6B**; **Supplementary Table 3**). With age, the percentage of CST-I, CST-III, and CST-IV-A gradually decreased, whereas the percentage of CST-IV-B gradually increased (**Figure 6A**; **Supplementary Table 3**).

## Discussion

This study was a large cross-sectional study that evaluated the relationship between vaginal microbiota and HPV infection. We tested the vaginal microbiota at the species level in Chinese women divided into three groups, including a healthy group, a HPV-infected group, and a CIN-diagnosed group. Significantly higher microbiota diversities were observed in the HPV-infected and CIN-diagnosed groups than in the healthy group. The bacterial communities sampled were classified into six CSTs based on differences in species composition and their relative abundances. We found that the CSTs were different in each group of women, and there may be a relationship between age and HPV infection.

Only four out of 356 women (1.12%) were classed under CST-II or CST-V, which are dominated by *L. gasseri* and *L. jensenii*. This reflects the findings of previous studies that *L. gasseri–*dominated cluster was usually dominated as 5% to 30%, whereas it was only 0.56% in our study (Ravel et al., 2011; Di Paola et al., 2017; Gosmann et al., 2017; Brotman et al., 2018; Caselli et al., 2020). This suggests that Chinese women may have a deficiency of *L. gasseri* or their vaginal microenvironment may not be suitable for its growth. Our results were also consistent with another Chinese study. Previous studies have indicated that *L. gasseri* might potentially be beneficial for HPV clearance (Brotman et al., 2014; Gosmann et al., 2017; Mitra et al., 2020). The lack of *L. gasseri–*dominated clusters suggests that Chinese women may have a weaker ability to clear HPV infection compared with those in other countries.

In all three groups, the proportions of CST-III and CST-IV-B were gradually increased, whereas the proportions of CST-I and CST-IV-A were gradually decreased. Previous studies indicated that domination with *L. iners* was an intermediate state that changed to *L. crispatus* domination when the vaginal environment became better and to non-*Lactobacillus* spp. domination when it became worse. CST-IV-B was considered a risk factor for persistent HPV infection because SCFAs, which destroy the vaginal mucosa, are secreted. The changes in CST-III and CST-IV-B clusters in our results suggest that the balance of VMB may be disrupted in the HPV and CIN groups. HPV infection may break the healthy vaginal microenvironment and build it suitable for the growth of pathogenic bacteria. *L. crispatus* forms a film on the surface of vaginal mucosa and secretes lactic acid to inhibit pathogenic bacteria. Its decrease indicates that the protective effect of VMB in the vagina is downregulated.

According to the logistic regression analysis, some factors including age, contraception, CST types, number of pregnancy, number of sexual partners, and IUD implant have relationship with HPV infection. Because the age was a continuous variable, we chose it and we wanted to find the trend with the increasing of age. The possibility of HPV infection, cervical lesions, and proportion of CST-IV-B gradually increased with age, but other CST clusters gradually decreased. This suggests that the status of VMB in Chinese women became worse with age. Factors such as reduced *Lactobacillus* spp. result in a weakened VMB homeostasis and a higher risk of HPV infection and growth of pathogenic bacteria. In addition, with the logistic regression analysis, during so many factors, only HPV infection was the risk factor for CIN. We wanted to find out the relationship between HPV infection and CIN, and we did not have a positive result.

To understand the reasons for *L. gasseri* deficiency in Chinese women, it is necessary to study both genetic and social factors by exploring the differences in gene expression in their reproductive tract and their unique living, eating, and cleaning habits. We also need protracted studies that track the average time Chinese women clear HPV infection or develop cervical lesions. This could be compared with studies in other countries and regions to examine if *L. gasseri* deficiency in Chinese women delays HPV infection clearance and promotes cervical lesions. If true, then they are possibly at higher risk of HPV infection than women in other regions, thus making HPV vaccination more important. The promotion of the HPV preventive vaccine in China came 10 years later than that in other countries. It is not included in China’s medical insurance, so there is no official vaccination rate available. Because of the possible delayed HPV infection clearance in Chinese women, it is necessary to be fully vaccinated against HPV. Further studies should explore the preventive effect of the vaccine in Chinese women.

Aging is a large research area associated with a variety of factors including decreased hormone levels, metabolic changes, and nutrient loss. In addition to the continuous variable of age, other factors also played a role in HPV infection. Non-use of contraception was a risk factor, whereas CST, pregnancy, number of sexual partners, and IUD were protective factors. In the research of CIN, HPV infection was the only risk factor and other factors did not have effect in CIN. Further research is needed on the mechanism between increased HPV infection and age. Previous studies focused on specific physiological stages such as pregnancy, perimenopause, and adolescence, but our research covered all women of reproductive age. It was a cross-sectional study, lacking long follow-up and mechanism research.

Chinese scholars started to study VMB later than foreign scholars. At present, studies are still focused on the stage of phenomenon observation, with a small amount of data from mostly cross-sectional studies. Further research can be extended to gene expression, epidemiological statistics, and mechanism studies. The effect of HPV preventive vaccination on the cervical health of Chinese women is also worth studying. Although the mechanism of aging is complex and there is no consensus, its effect on HPV infection and VMB is worth studying as well.

In conclusion, our research brings essential comparable data to the Chinese vaginal microbiome research. We found that the lack of *L. gasseri* domination in Chinese women was different compared with studies in other countries. The large sample size showed the changing trend of CST subtypes with HPV infection and cervical lesion progression. Aging was a risk factor of HPV infection, but there is a need to study the mechanism of HPV infection and cervical lesion development.

## Data Availability Statement

The datasets presented in this study can be found in online repositories. The names of the repository/repositories and accession number(s) can be found below: NCBI; PRJNA787754.

## Ethics Statement

The studies involving human participants were reviewed and approved by Aviation General Hospital Ethics Committee. The patients/participants provided their written informed consent to participate in this study.

## Author Contributions

YiZ: experiment design, sample collection, experimental operation, and article writing. XX: sample collection and experimental operation. LY, XS, MM, and LX: sample collection. JP: partly data analysis. YoZ: provision of laboratory. PL and GW: experiment guidance. GG: experiment design and experiment guidance. All authors contributed to the article and approved the submitted version.

## Conflict of Interest

The authors declare that the research was conducted in the absence of any commercial or financial relationships that could be construed as a potential conflict of interest.

## Publisher’s Note

All claims expressed in this article are solely those of the authors and do not necessarily represent those of their affiliated organizations, or those of the publisher, the editors and the reviewers. Any product that may be evaluated in this article, or claim that may be made by its manufacturer, is not guaranteed or endorsed by the publisher.

## References

[B1] Agostinis C. Mangogna A. Bossi F. Ricci G. Kishore U. Bulla R. (2019). Uterine Immunity and Microbiota: A Shifting Paradigm. Front. Immunol. 10. doi: 10.3389/fimmu.2019.02387 PMC6811518 31681281

[B2] Amabebe E. Anumba D. O. C. (2018). The Vaginal Microenvironment: The Physiologic Role of Lactobacilli. Front. Med. (Lausanne). 5. doi: 10.3389/fmed.2018.00181 PMC6008313 29951482

[B3] Andrade Pessoa Morales J. Marconi C. El-Zein M. Ravel J. da Silva Pinto G. V. Silveira R.. (2021). Vaginal Microbiome Components as Correlates of Cervical Human Papillomavirus Infection. J. Infect. Dis. 28, jiab547. doi: 10.1093/infdis/jiab547 34718662

[B4] Avilés-Jiménez F. Yu G. Torres-Poveda K. Madrid-Marina V. Torres J. (2017). On the Search to Elucidate the Role of Microbiota in the Genesis of Cancer: The Cases of Gastrointestinal and Cervical Cancer. Arch. Med. Res. 48 (8), 754–765. doi: 10.1016/j.arcmed.2017.11.008 29203054

[B5] Borgdorff H. Gautam R. Armstrong S. D. Xia D. Ndayisaba G. F. van Teijlingen N. H.. (2016). Cervicovaginal Microbiome Dysbiosis is Associated With Proteome Changes Related to Alterations of the Cervicovaginal Mucosal Barrier. Mucosal Immunol. 9 (3), 621–633. doi: 10.1038/mi.2015.86 26349657

[B6] Borgogna J. C. Shardell M. D. Santori E. K. Nelson T. M. Rath J. M. Glover E. D.. (2020). Authors' Reply Re: The Vaginal Metabolome and Microbiota of Cervical HPV-Positive and HPV-Negative Women: A Cross-Sectional Analysis. BJOG 127 (6), 773–774. doi: 10.1111/1471-0528.16148 PMC10281050 32154972

[B7] Boskey E. R. Cone R. A. Whaley K. J. Moench T. R. (2001). Origins of Vaginal Acidity: High D/L Lactate Ratio is Consistent With Bacteria Being the Primary Source. Hum. Reprod. 16 (9), 1809–1813. doi: 10.1093/humrep/16.9.1809 11527880

[B8] Brotman R. M. Shardell M. D. Gajer P. Fadrosh D. Chang K. Silver M. I.. (2018). Association Between the Vaginal Microbiota, Menopause Status, and Signs of Vulvovaginal Atrophy. Menopause 25 (11), 1321–1330. doi: 10.1097/GME.0000000000001236 30358729

[B9] Brotman R. M. Shardell M. D. Gajer P. Tracy J. K. Zenilman J. M. Ravel J.. (2014). Interplay Between the Temporal Dynamics of the Vaginal Microbiota and Human Papillomavirus Detection. J. Infect. Dis. 210 (11), 1723–1733. doi: 10.1093/infdis/jiu330 24943724 PMC4296189

[B10] Caselli E. D'Accolti M. Santi E. Soffritti I. Conzadori S. Mazzacane S.. (2020). Vaginal Microbiota and Cytokine Microenvironment in HPV Clearance/Persistence in Women Surgically Treated for Cervical Intraepithelial Neoplasia: An Observational Prospective Study. Front. Cell Infect. Microbiol. 10. doi: 10.3389/fcimb.2020.540900 PMC7676899 33251154

[B11] Chao X. P. Sun T. T. Wang S. Fan Q. B. Shi H. H. Zhu L.. (2019). Correlation Between the Diversity of Vaginal Microbiota and the Risk of High-Risk Human Papillomavirus Infection. Int. J. Gynecol. Cancer. 29 (1), 28–34. doi: 10.1136/ijgc-2018-000032 30640680

[B12] Chen Y. Hong Z. Wang W. Gu L. Gao H. Qiu L.. (2019). Association Between the Vaginal Microbiome and High-Risk Human Papillomavirus Infection in Pregnant Chinese Women. BMC Infect. Dis. 19 (1), 677. doi: 10.1186/s12879-019-4279-6 31370796 PMC6669982

[B13] Chen Y. Qiu X. Wang W. Li D. Wu A. Hong Z.. (2020). Human Papillomavirus Infection and Cervical Intraepithelial Neoplasia Progression Are Associated With Increased Vaginal Microbiome Diversity in a Chinese Cohort. BMC Infect. Dis. 20 (1), 629. doi: 10.1186/s12879-020-05324-9 32842982 PMC7449047

[B14] Delgado-Diaz D. J. Tyssen D. Hayward J. A. Gugasyan R. Hearps A. C. Tachedjian G. (2020). Distinct Immune Responses Elicited From Cervicovaginal Epithelial Cells by Lactic Acid and Short Chain Fatty Acids Associated With Optimal and Non-Optimal Vaginal Microbiota. Front. Cell Infect. Microbiol. 9. doi: 10.3389/fcimb.2019.00446 PMC6965070 31998660

[B15] Di Paola M. Sani C. Clemente A. M. Iossa A. Perissi E. Castronovo G.. (2017). Characterization of Cervico-Vaginal Microbiota in Women Developing Persistent High-Risk Human Papillomavirus Infection. Sci. Rep. 7 (1), 10200. doi: 10.1038/s41598-017-09842-6 28860468 PMC5579045

[B16] Di Pietro M. Filardo S. Falasca F. Turriziani O. Sessa R. (2017). Infectious Agents in Atherosclerotic Cardiovascular Diseases Through Oxidative Stress. Int. J. Mol. Sci. 18 (11), 2459. doi: 10.3390/ijms18112459 PMC5713425 29156574

[B17] Gajer P. Brotman R. M. Bai G. Sakamoto J. Schütte U. M. Zhong X.. (2012). Temporal Dynamics of the Human Vaginal Microbiota. Sci. Transl. Med. 4 (132), 132ra52. doi: 10.1126/scitranslmed.3003605 PMC3722878 22553250

[B18] Gosmann C. Anahtar M. N. Handley S. A. Farcasanu M. Abu-Ali G. Bowman B. A.. (2017). Lactobacillus-Deficient Cervicovaginal Bacterial Communities Are Associated With Increased HIV Acquisition in Young South African Women. Immunity 46 (1), 29–37. doi: 10.1016/j.immuni.2016.12.013 28087240 PMC5270628

[B19] Li Y. Yu T. Yan H. Li D. Yu T. Yuan T.. (2020). Vaginal Microbiota and HPV Infection: Novel Mechanistic Insights and Therapeutic Strategies. Infect. Drug Resist. 13, 1213–1220. doi: 10.2147/IDR.S210615 32431522 PMC7198448

[B20] Mei L. Wang T. Chen Y. Wei D. Zhang Y. Cui T.. (2022). Dysbiosis of Vaginal Microbiota Associated With Persistent High-Risk Human Papilloma Virus Infection. J. Transl. Med. 20 (1), 12. doi: 10.1186/s12967-021-03201-w 34980148 PMC8722078

[B21] Mitra A. MacIntyre D. A. Marchesi J. R. Lee Y. S. Bennett P. R. Kyrgiou M. (2016). The Vaginal Microbiota, Human Papillomavirus Infection and Cervical Intraepithelial Neoplasia: What do We Know and Where are We Going Next? Microbiome 4 (1), 58. doi: 10.1186/s40168-016-0203-0 27802830 PMC5088670

[B22] Mitra A. MacIntyre D. A. Ntritsos G. Smith A. Tsilidis K. K. Marchesi J. R.. (2020). The Vaginal Microbiota Associates With the Regression of Untreated Cervical Intraepithelial Neoplasia 2 Lesions. Nat. Commun. 11 (1), 1999. doi: 10.1038/s41467-020-15856-y 32332850 PMC7181700

[B23] Moodley M. Moodley J. Chetty R. Herrington C. S. (2003). The Role of Steroid Contraceptive Hormones in the Pathogenesis of Invasive Cervical Cancer: A Review. Int. J. Gynecol. Cancer. 13 (2), 103–110. doi: 10.1046/j.1525-1438.2003.13030.x 12657108

[B24] Murall C. L. Rahmoun M. Selinger C. Baldellou M. Bernat C. Bonneau M.. (2019). Natural History, Dynamics, and Ecology of Human Papillomaviruses in Genital Infections of Young Women: Protocol of the PAPCLEAR Cohort Study. BMJ Open 9 (6), e025129. doi: 10.1136/bmjopen-2018-025129 PMC6576111 31189673

[B25] Norenhag J. Du J. Olovsson M. Verstraelen H. Engstrand L. Brusselaers N. (2020). The Vaginal Microbiota, Human Papillomavirus and Cervical Dysplasia: A Systematic Review and Network Meta-Analysis. BJOG 127 (2), 171–180. doi: 10.1111/1471-0528.15854 31237400

[B26] Piersma S. J. (2011). Immunosuppressive Tumor Microenvironment in Cervical Cancer Patients. Cancer Microenviron. 4 (3), 361–375. doi: 10.1007/s12307-011-0066-7 21626415 PMC3234326

[B27] Ravel J. Gajer P. Abdo Z. Schneider G. M. Koenig S. S. McCulle S. L.. (2011). Vaginal Microbiome of Reproductive-Age Women. Proc. Natl. Acad. Sci. U S A 108 Suppl 1 (Suppl 1), 4680–4687. doi: 10.1073/pnas.1002611107 20534435 PMC3063603

[B28] Wu M. Gao J. Wu Y. Li Y. Chen Y. Zhao F.. (2020). Characterization of Vaginal Microbiota in Chinese Women With Cervical Squamous Intra-Epithelial Neoplasia. Int. J. Gynecol. Cancer. 30 (10), 1500–1504. doi: 10.1136/ijgc-2020-001341 32499394

[B29] Zhai Q. Ren T. Fu Y. Zhang Z. Li L. Li Y.. (2020). [Characteristics of Cervical Microecology in Late Reproductive-Age Women With Different Grades of Cervical Lesions]. Nan Fang Yi Ke Da Xue Xue Bao 40 (12), 1768–1775. doi: 10.12122/j.issn.1673-4254.2020.12.11 33380398 PMC7835688

