# Supplementary material for: Vaginal Microbiota Changes Caused by HPV Infection in Chinese Women
Source: Front Cell Infect Microbiol. 2022 Jun 21;12:814668. doi: 10.3389/fcimb.2022.814668 (PMC9253274; doi:10.3389/fcimb.2022.814668)
Supplement: Supplementary file 1 [file DataSheet_1.docx]

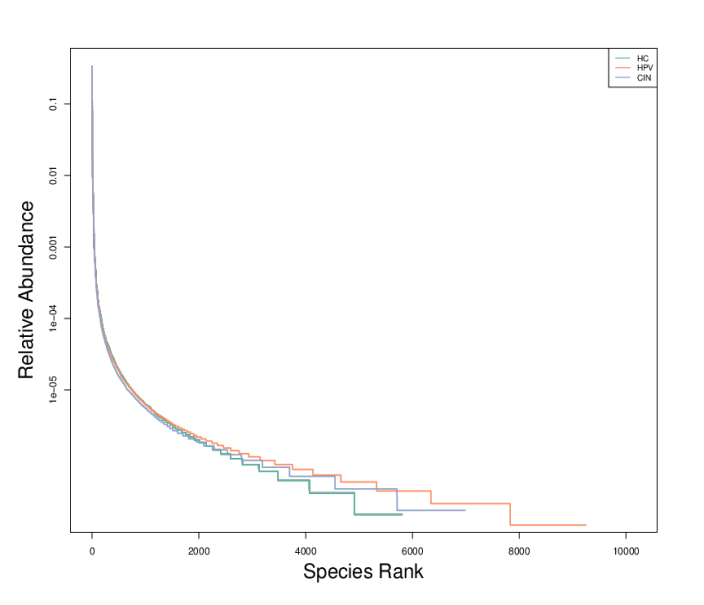


**A**

**Supplementary Figure 1: Microbial alpha diversity analysis based on Rank Abundance Curve.**

**A)** Rank abundance curve of groups.


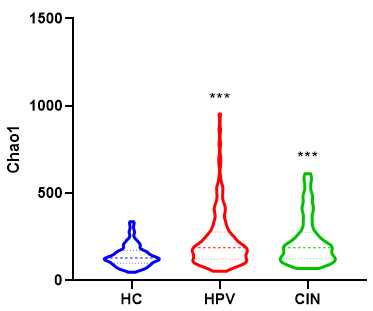

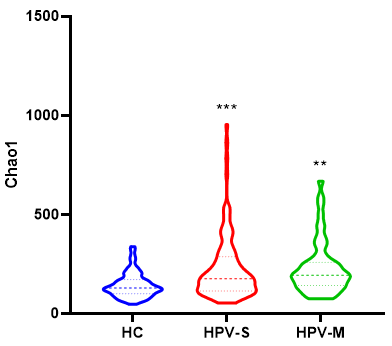


**B**

**A**

**D**

**C**


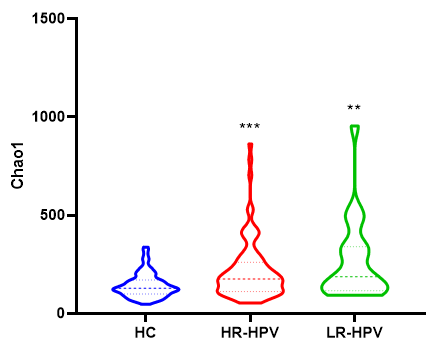

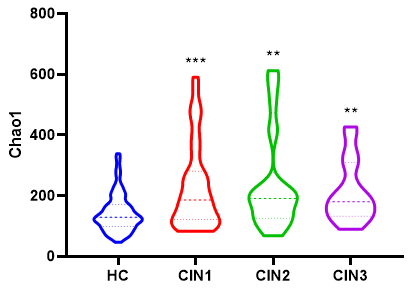


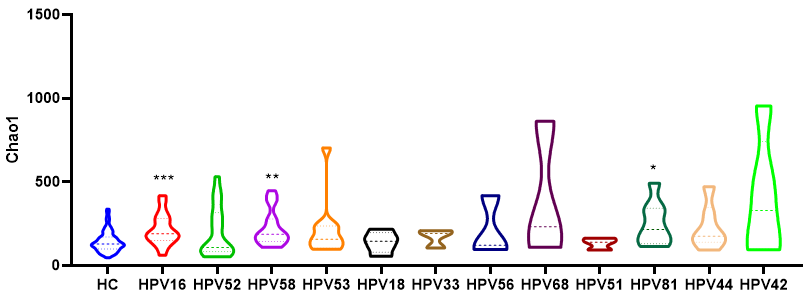


**E**

**Supplementary Figure 2: Microbial alpha diversity analysis based on Chao1 analysis. A)** Microbiota diversity was significantly higher in HPV infected group and CIN diagnosed group than in the healthy control group. (Wilcoxon one-side test; ***p<0.0001). **B)** Microbiota diversity was significantly higher in HPV-S group and HPV-M group than in healthy control group (Wilcoxon one-side test; **p<0.005, ***p<0.0001). **C)** Microbiota diversity was significantly higher in HR-HPV group and LR-HPV group than in healthy control group (Wilcoxon one-side test; **p<0.005, ***p<0.0001). **D)** CIN1, 2, 3 diagnosed groups had significantly higher microbiota diversity than healthy control group (Wilcoxon one-side test; **p<0.005, ***p<0.0001). **E)** Microbiota diversity among participants infected with different HPV types in comparison with healthy women. Significantly higher mucrobiota diversity was observed from women infected with HPV16, 58 and 81 compared to the healthy women (Wilcoxon one-side test; *p<0.05, **p<0.005, ***p<0.0001).


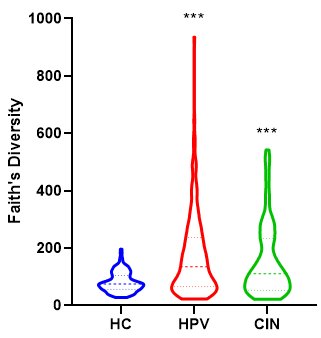

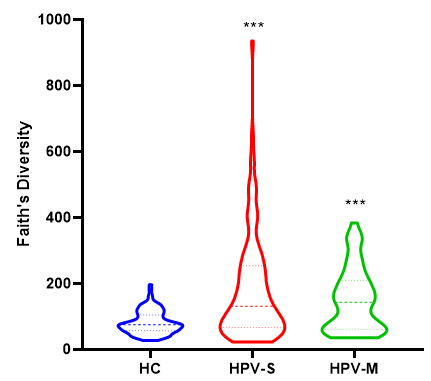


**B**

**A**

**D**

**C**


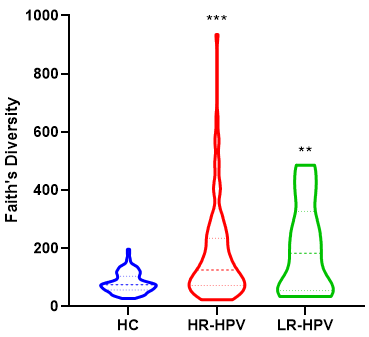

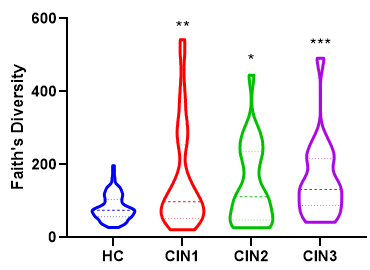


**E**


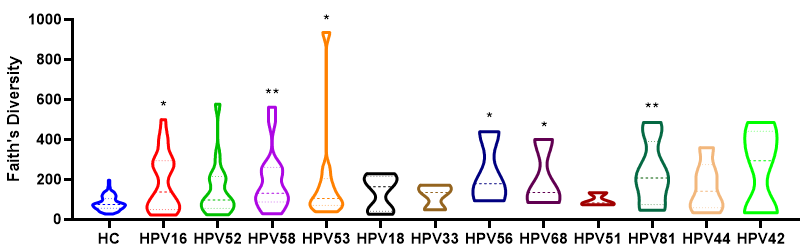


**Supplementary Figure 3: Microbial alpha diversity analysis based on Faith’s phylogenetic diversity. A)** Microbiota diversity was significantly higher in HPV infected group and CIN diagnosed group than in the healthy control group. (Wilcoxon one-side test; ***p<0.0001). **B)** Microbiota diversity was significantly higher in HPV-S group and HPV-M group than in healthy control group (Wilcoxon one-side test; ***p<0.0001). **C)** Microbiota diversity was significantly higher in HR-HPV group and LR-HPV group than in healthy control group (Wilcoxon one-side test; **p<0.005, ***p<0.0001). **D)** CIN1, 2, 3 diagnosed groups had significantly higher microbiota diversity than healthy control group (Wilcoxon one-side test; *p<0.05, **p<0.005, ***p<0.0001). **E)** Microbiota diversity among participants infected with different HPV types in comparison with healthy women. Significantly higher mucrobiota diversity was observed from women infected with HPV16, 58 53, 56, 68 and 81 compared to the healthy women (Wilcoxon one-side test; *p<0.05, **p<0.005).


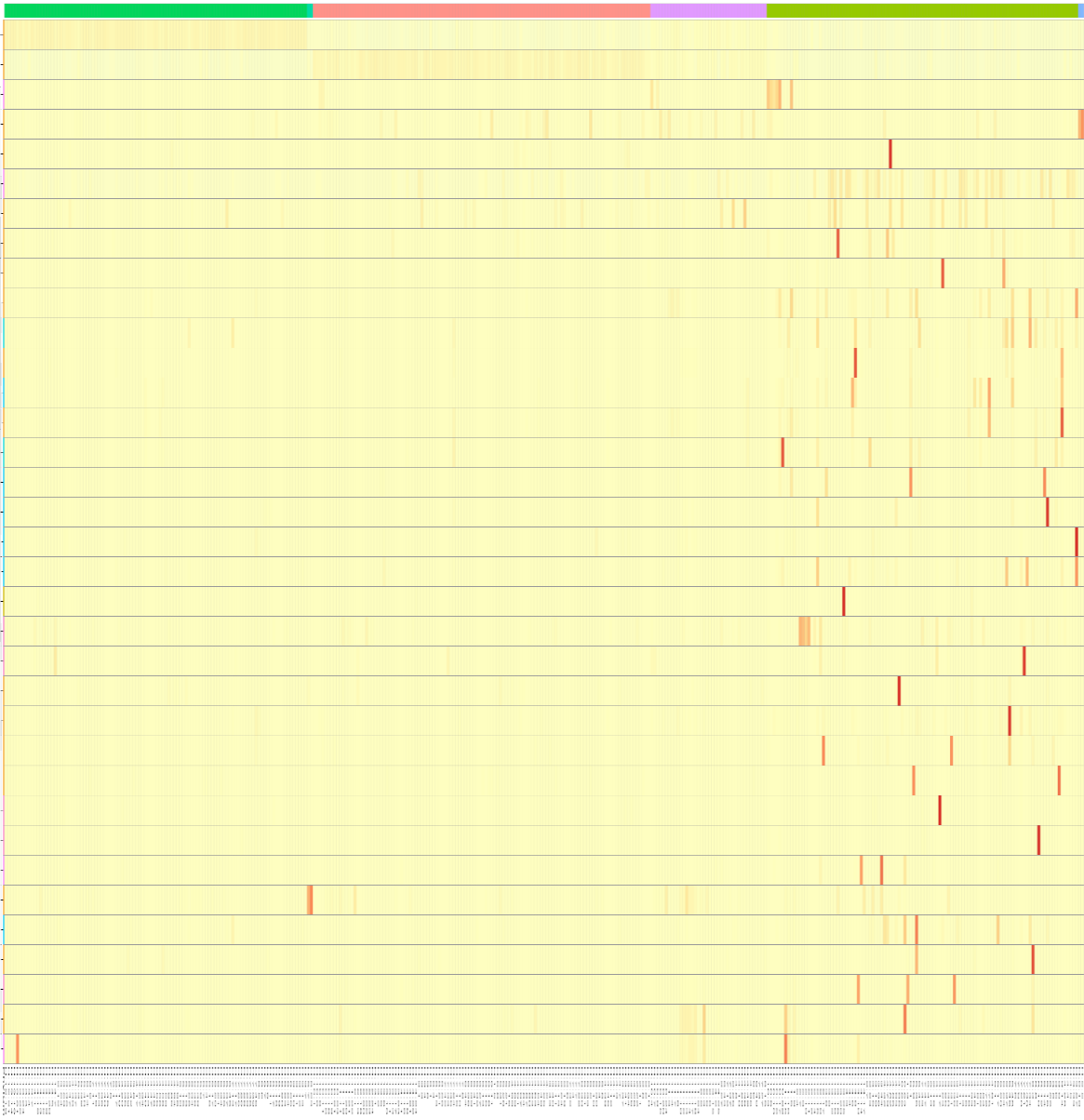

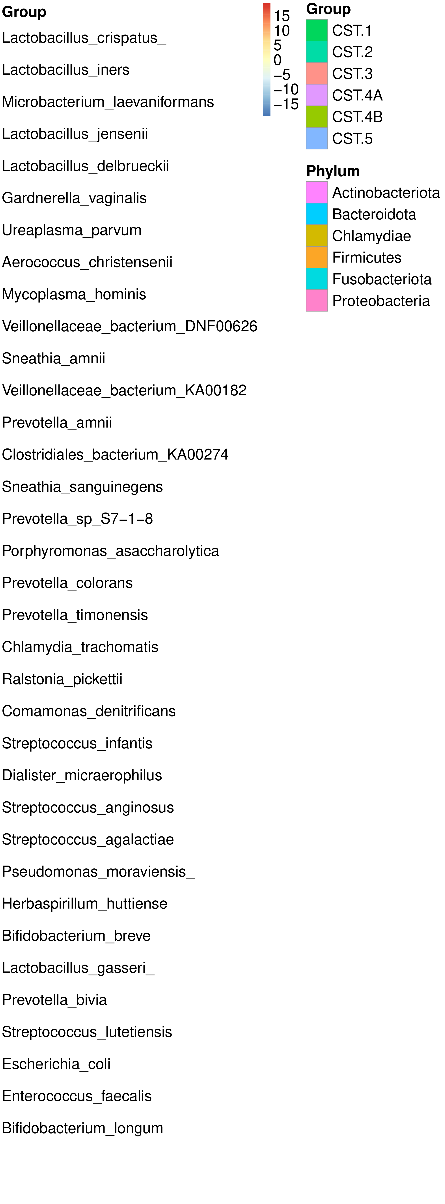


**A**

**Supplementary Figure 4: Samples were classified by heatmap. A)** Heatmap of log10-transformed proportions of microbial taxa found in the vaginal bacterial communities of 356 women.


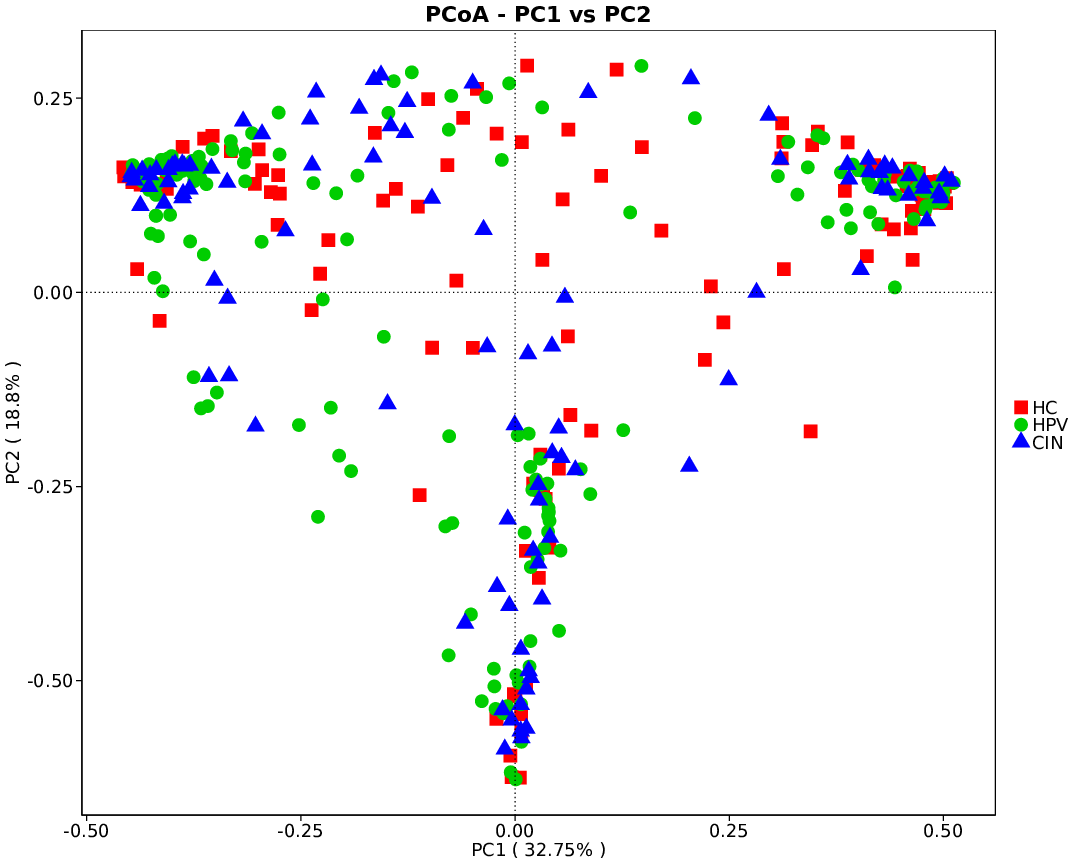


**A**

**B**


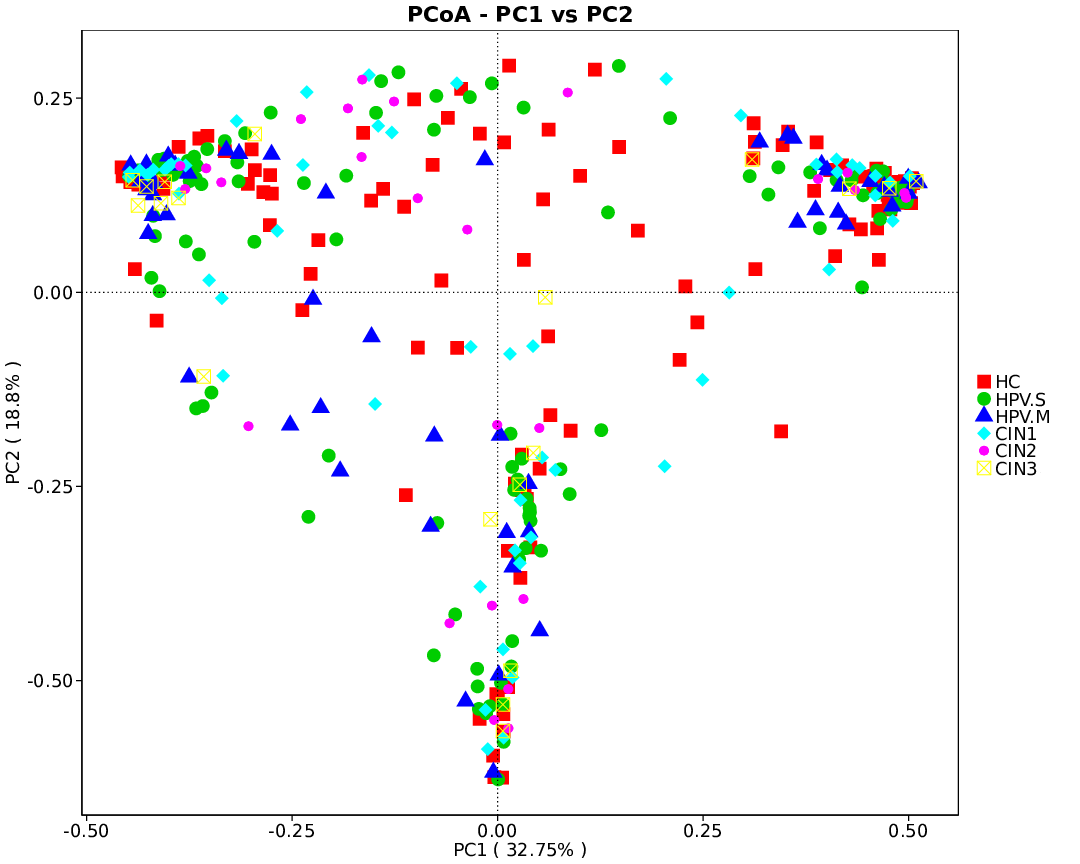


**Supplementary Figure 5:** **Principal coordinates analysis (PCoA) of microbiota data** **based on Bray-Curtis distance. A) and B)** PCoA plot with the samples colores according to HPV infectious and CIN diagnosed status. No clear separation was observed among healthy samples, HPV infected samples and CIN samples.

**
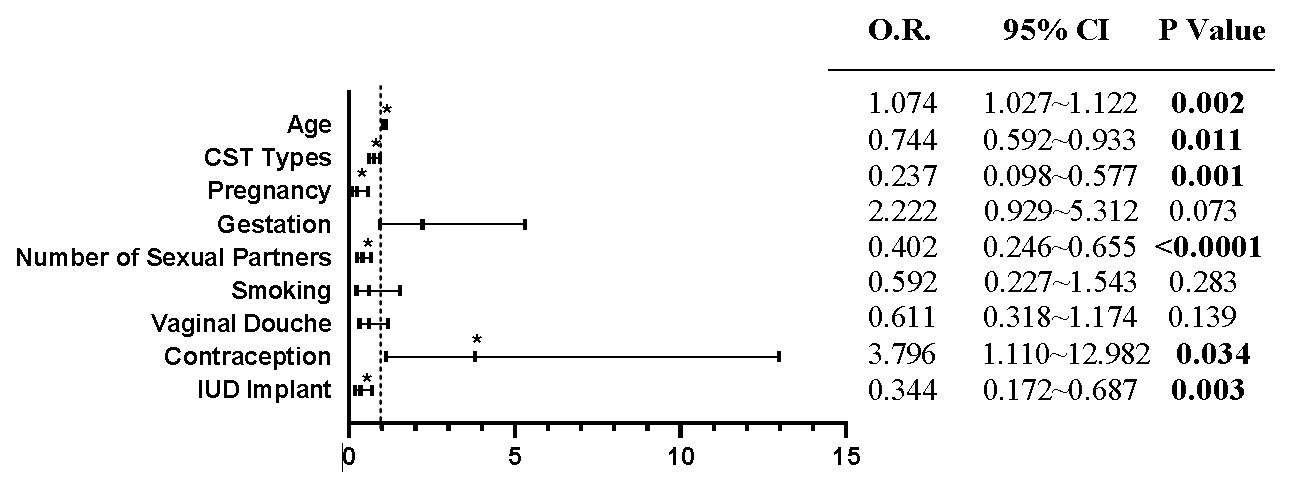
**

**A**

**
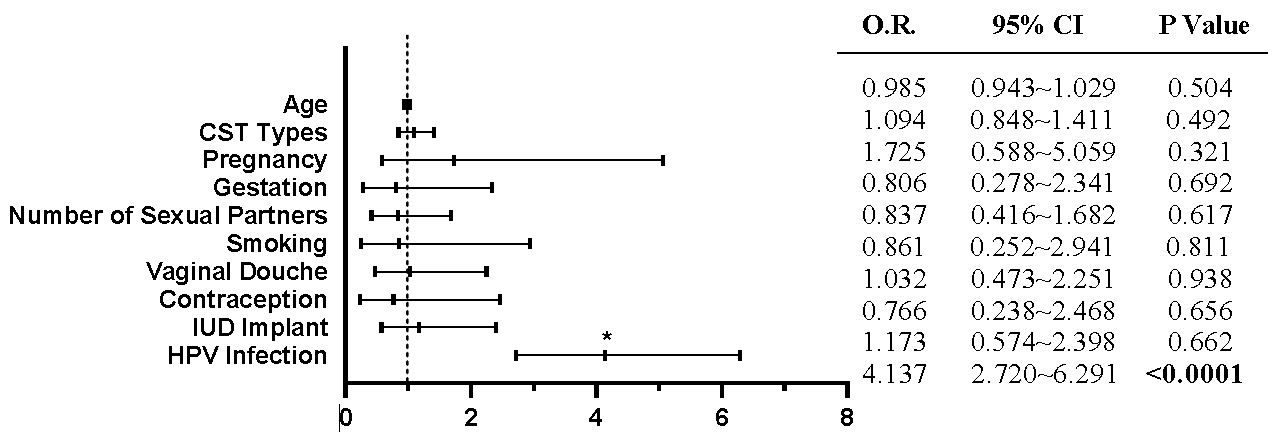
**

**B**

**Supplementary Figure 6:** **Odds ratio of possible risk factors for HPV/CIN Using Logistic regression. A)** Odds ratio of possible risk factors for HPV; **B)** Odds ratio of possible risk factors for CIN. Bars represented 95% confidence intervals. O.R.: odds ratio, CI: confidence interval. * *P* < .05

**Supplementary Table 1: The characteristics of HPV infection types in all samples**

| **HPV Types** | **N** | **Percentage (%)** |  |
| --- | --- | --- | --- |
| **HPV(-)** | 113 | 31.74% | |
| **HPV16** | 40 | 11.24% | 42.70% |
| **HPV52** | 22 | 6.18% |  |
| **HPV58** | 22 | 6.18% |  |
| **HPV53** | 10 | 2.81% |  |
| **HPV18** | 8 | 2.25% |  |
| **HPV81** | 8 | 2.25% |  |
| **HPV33** | 6 | 1.69% |  |
| **HPV44** | 6 | 1.69% |  |
| **HPV51** | 5 | 1.40% |  |
| **HPV42** | 5 | 1.40% |  |
| **HPV68** | 4 | 1.12% |  |
| **HPV59** | 3 | 0.84% |  |
| **HPV56** | 3 | 0.84% |  |
| **HPV31** | 2 | 0.56% |  |
| **HPV43** | 2 | 0.56% |  |
| **HPV66** | 2 | 0.56% |  |
| **HPV82** | 1 | 0.28% |  |
| **HPV39** | 1 | 0.28% |  |
| **HPV73** | 1 | 0.28% |  |
| **HPV61** | 1 | 0.28% |  |
| **2HR-HPV** | 43 | 12.08% | 25.56% |
| **3HR-HPV** | 11 | 3.09% |  |
| **4HR-HPV** | 2 | 0.56% |  |
| **HR-HPV+LR-HPV** | 33 | 9.27% |  |
| **2LR-HPV** | 2 | 0.56% |  |
| **Total** | 356 | 100% | |

**Supplementary Table 2: The characteristics of HPV infection types in HPV positive samples including HPV group and CIN group**

| **HPV types** | **HPV group (%)** | **CIN group (%)** | **HPV+CIN group (%)** | |
| --- | --- | --- | --- | --- |
| **16** | 20(12.58) | 20(23.81) | 40(16.46) | 152(62.55) |
| **52** | 16(10.06) | 6(7.14) | 22(9.05) |  |
| **58** | 15(9.43) | 7(8.33) | 22(9.05) |  |
| **53** | 10(6.29) | 0(0.00) | 10(4.12) |  |
| **18** | 5(3.14) | 3(3.57) | 8(3.29) |  |
| **81** | 8(5.03) | 0(0.00) | 8(3.29) |  |
| **33** | 3(1.89) | 3(3.57) | 6(2.47) |  |
| **44** | 6(3.77) | 0(0.00) | 6(2.47) |  |
| **51** | 3(1.89) | 2(2.38) | 5(2.06) |  |
| **42** | 5(3.14) | 0(0.00) | 5(2.06) |  |
| **68** | 3(1.89) | 1(1.19） | 4(1.65) |  |
| **59** | 2(1.26) | 1(1.19） | 3(1.23) |  |
| **56** | 3(1.89) | 0(0.00) | 3(1.23) |  |
| **31** | 2(1.26) | 0(0.00） | 2(0.82) |  |
| **43** | 1(0.63) | 1(1.19） | 2(0.82) |  |
| **66** | 2(1.26) | 0(0.00) | 2(0.82) |  |
| **82** | 1(0.63) | 0(0.00) | 1(0.41) |  |
| **39** | 1(0.63) | 0(0.00) | 1(0.41) |  |
| **73** | 0(0.00) | 1(1.19） | 1(0.41) |  |
| **61** | 1(0.63) | 0(0.00) | 1(0.41) |  |
| **2HR-HPV** | 24(15.09) | 19(22.62) | 43(17.70) | 91(37.45) |
| **3HR-HPV** | 7(4.40) | 4(4.76) | 11(4.53) |  |
| **4HR-HPV** | 0(0.00) | 2(2.38) | 2(0.82) |  |
| **HR-HPV+LR-HPV** | 19(11.95) | 14(16.67) | 33(13.58) |  |
| **2LR-HPV** | 2(1.26) | 0(0.00) | 2(0.82) |  |
| **Total** | 159 | 84 | 243 | 243 |

**Supplementary Table 3:** Characteristics of samples at different age stages.

|  | **20-29** | **30-39** | **40-49** | **50-59** | **60-69** | **p-value** |
| --- | --- | --- | --- | --- | --- | --- |
|  | **(years)** | **(years)** | **(years)** | **(years)** | **(years)** | **(**χ2 test) |
| **HC** | 27(38.57%) | 67(40.61%) | 11(16.18%) | 7(17.50%) | 1(7.69%) | <0.0001 |
| **HPV** | 31(44.29%) | 61(36.97%) | 34(50.00%) | 26(65.00%) | 7(53.85%) |  |
| **CIN** | 12(17.14%) | 37(22.42%) | 23(33.82%) | 7(17.50%) | 5(38.46%) |  |
| **CST-I** | 26(37.14%) | 55(33.33%) | 17(25.00%) | 3(7.50%) | 1(7.69%) | <0.0001 |
| **CST-II** | 0(0.00%) | 1(0.61%) | 0(0.00%) | 1(2.50%) | 0(0.00%) |  |
| **CST-III** | 27(38.57%) | 49(29.70%) | 25(36.76%) | 9(22.50%) | 1(7.69%) |  |
| **CST-IV-A** | 6(8.57%) | 23(13.94%) | 10(14.71%) | 1(2.50%) | 0(0.00%) |  |
| **CST-IV-B** | 11(15.71%) | 35(21.21%) | 16(23.53%) | 26(65.00%) | 11(84.62%) |  |
| **CST-V** | 0(0.00%) | 2(1.21%) | 0(0.00%) | 0(0.00%) | 0(0.00%) |  |

**Supplementary Table 4: Special HPV types with CIN degree**

|  | **CIN1** | **CIN2** | **CIN3** | **p-value (χ2 test）** |
| --- | --- | --- | --- | --- |
| **HPV-S** | 24 | 14 | 7 | 0.845 |
| **HPV-M** | 23 | 10 | 6 |  |
| **HPV16** | 11 | 6 | 3 | 0.791 |
| **HPV52** | 4 | 2 | 0 |  |
| **HPV58** | 2 | 4 | 1 |  |
| **HPV18** | 2 | 1 | 0 |  |
| **HPV33** | 1 | 1 | 1 |  |
